# The Effectiveness and Practicality of a Novel Barrier Membrane for the Open Window in Maxillary Sinus Augmentation with a Lateral Approach, with Risk Indicators for Bone Graft Displacement and Bone Height Decrease: A Prospective Study in Humans

**DOI:** 10.3390/bioengineering10101110

**Published:** 2023-09-22

**Authors:** Kikue Yamaguchi, Motohiro Munakata, Daisuke Sato, Yu Kataoka, Ryota Kawamata

**Affiliations:** 1 Department of Implant Dentistry, Showa University School of Dentistry, 2-1-1, Kita-senzoku, Ota-ku, Tokyo 1458515, Japan; munakata@dent.showa-u.ac.jp (M.M.); dsato.imp@dent.showa-u.ac.jp (D.S.); kawamata@kdu.ac.jp (R.K.); 2 Department of Dental Education, Showa University School of Dentistry, 1-8-5, Hatanodai, Shinagawa-ku, Tokyo 1428555, Japan; yu-kataoka@dent.showa-u.ac.jp; 3 Department of Biomaterials and Engineering, Showa University School of Dentistry, 1-8-5, Hatanodai, Shinagawa-ku, Tokyo 14228555, Japan

**Keywords:** sinus augmentation, lateral approach, barrier membrane, bone graft displacement, carbonate apatite

## Abstract

Maxillary sinus augmentation with a lateral approach (MSA) is a well-established treatment. In this prospective study, we evaluated risk factors for postoperative bone graft displacement and reported the clinical application of long-term resorbable L-lactic acid/-caprolactone (PLA/PCL) as a barrier membrane to cover the open window in the lateral wall in MSA. Twenty-four patients underwent MSA according to the relevant criteria; CT data obtained before and 1 week (1 w) and 5–6 months (5 m) post-MSA, bone height changes, bone height reduction rates at 1 w and 5 m post-MSA, bone graft displacement measurements, and risk factors were examined. All patients showed bone height increments (*p* < 0.005). However, no difference was observed between 1 w and 5 m post-MSA. Bone graft displacement was observed in eight patients; the reduction rate from 1 w to 5 m post-MSA was 8.38% ± 4.88%. Sex, septa, maxillary sinus floor–palatal bone distance, and maxillary sinus floor–maxillary ostium distance were associated with bone graft displacement (*p* < 0.05). The height from the maxillary sinus floor to the palatal bone and the sinus angle influenced the augmentation degree (*p* < 0.05). The PLA/PCL membrane is compared favorably with other membranes and may be useful as a barrier membrane for the MSA open window.

## 1. Introduction

In patients undergoing implant therapy for maxillary molar defects, the lack of existing bone height in the maxillary molars often prevents ideal implant placement. Maxillary sinus augmentation with a lateral approach (MSA) is performed to solve this problem. MSA has become an established treatment method for maxillary molars. Long-term prognosis data show good outcomes, with systematic reviews showing a high implant survival rate of 92–100%. No differences in implant survival rates have been observed based on graft material type (autograft, allograft, xenograft, alloplastic bone, or their combinations) or the initial bone height (less than 5 mm and 5 mm or greater) [1,2,3,4,5]. On the contrary, a range of complications has been reported, including intraoperative arterial injury, sinus membrane perforation, postoperative facial swelling and pain, graft infection, and consequent maxillary sinusitis [6].

Complications related to MSA include sinus membrane perforation, maxillary sinusitis, and graft material displacement. Sinus membrane perforation occurs in a relatively high proportion of patients (20–25%), and its development is influenced by anatomical characteristics such as thin sinus mucosa, residual bone height, narrow sinus angle, the presence of septa, and thick lateral walls. Additional factors contributing to perforation include the presence of smears, smoking habits, surgical techniques involving sinus floor instruments, and the surgeons’ expertise. Maxillary sinusitis following MSA occurs in approximately 4.2–8.4% of cases. This is associated with preoperative issues in the osteomeatal complex, intraoperative large membrane perforations, overfilling graft material, and mucosal swelling post-surgery. The incidence of such cases is high in the UK. Moreover, graft material displacement is caused by overfilling of the graft material and inadequate barrier membrane coverage at the lateral open window, which leads to reduced bone height due to MSA. Bacterial infection can cause facial pain or swelling and the previously mentioned maxillary sinusitis, often necessitating the removal of the graft material and the implant body. Placing a barrier membrane in the lateral open window has been shown to be important in solving problems caused by graft material displacement. This approach improves new bone formation rates and reduces the amount of residual graft material. However, the differences in effectiveness between different types of barrier membranes (resorbable or non-resorbable) and materials (titanium, expanded polytetrafluoroethylene [e-PTFE], PLLA–PGA copolymer, animal-derived collagen, etc.) have not been investigated to date [7,8,9,10].

The placement of a barrier membrane has been reported to promote bone formation and increase the implant survival rate because the barrier membrane covering the open window in MSA can prevent fibroblast invasion from connective tissue during wound healing and promote bone regeneration around the bone graft material [11,12,13,14,15,16]. However, the barrier membrane has also been reported to decrease blood supply to the bone graft material and increase the risk of infection [16,17,18,19]. Barrier membranes are categorized as resorbable or non-resorbable. While non-resorbable membranes can reliably block fibroblast invasion during the bone regeneration period, allowing for osteogenesis, another invasive surgery is needed to remove the membrane, which may lead to complications such as exposure and infection due to lack of blood flow [6]. On the other hand, resorbable membranes do not need to be removed because the body absorbs them, and most of these membranes are made from collagen derived from animals such as pigs and cows. However, the prediction of the degradation and resorption rate of membranes produced from collagen is complex, and it remains unclear whether they maintain a defense mechanism that prevents cell invasion [14]. Regarding the need for a covering membrane, Tarnow et al. [20] reported that implant survival was 100% when covered with an expanded e-PTFE membrane and 93% when not covered. Tawil et al. [21] also reported a much higher success rate when the implant was covered with a collagen membrane than when it was not covered. Ohayon et al. [22] studied bone graft material displacement from the open window with and without a barrier membrane. They reported that a barrier membrane was useful. That bone graft displacement from the open window correlated with postoperative thickening of the maxillary sinus mucosa and postoperative complications such as infection, intraoral pain, and facial swelling. These findings indicate that barrier membranes should have selective permeability, such as resorbability, slow absorption rate, and maintaining vascular growth within the membrane.

In the present study, we prospectively evaluated the effectiveness of L-lactic acid/ε-caprolactone (PLA/PCL), a long-term resorbable guided bone regeneration membrane, as a barrier membrane for lateral approach maxillary sinus augmentation and examined the risk factors associated with postoperative bone graft displacement and reduced bone height.

## 2. Materials and Methods

### 2.1. Research Design and Ethical Approval

**Figure 1:**
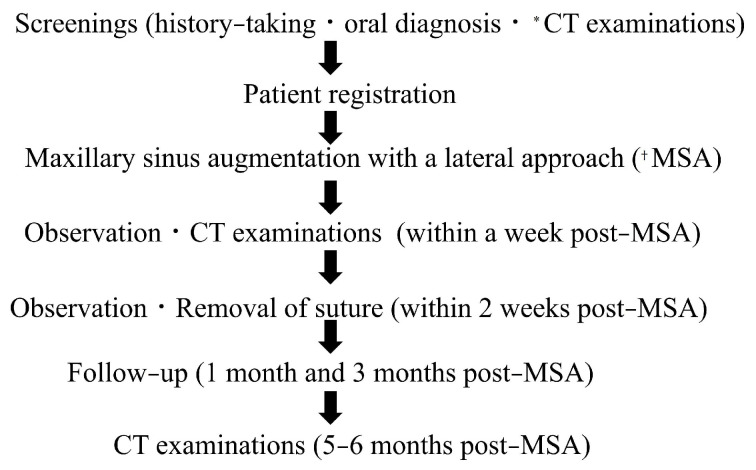
The study protocol. All maxillary sinus augmentation is performed in a 2stage procedure. * CT, computed tomography; ^†^ MSA, maxillary sinus augmentation with a lateral approach.

This prospective study was conducted at Showa University Dental Hospital Implant Center. The clinical trial, including patient recruitment, was conducted from October 2018 to May 2022, according to the protocol in Figure 1. The study participants were non-smoking patients aged 20–65 years with maxillary molar defects requiring two-stage MSA with a residual bone height of ≤4 mm. Table 1 shows the inclusion and exclusion criteria.

This study was approved by the Ethics Committee of SHOWA University Clinical Research Review Board (approval number jRCTs032190123 and 19 October 2019 approval) and by the Institutional Review Board of Showa University Dental Hospital (approval No. SUDH0043). It was conducted in accordance with the Declaration of Helsinki. All participants gave written informed consent for this study. Informed consent was obtained from all subjects involved in the study.

### 2.2. Graft Material

The artificial bone substitute was carbonate apatite (Cytrans Granules^®^, GC Corporation, Tokyo, Japan: hereafter CO3Ap). Carbonated apatite is synthesized by a dissolution–precipitation reaction and has the chemical formula Ca10-a(CO3)b(PO4)6-c. CO3Ap contains 6–9% carbonate by weight in an apatite crystal structure, and the particle size (short diameter) of CO3Ap ranges from 600 to 1000 μm. Carbonate apatite is thermodynamically stable in a neutral environment in vivo. In contrast, in a weakly acidic environment (pH 5), where osteoclasts form, it becomes unstable and dissolves, exhibiting excellent bone replacement properties [23].

### 2.3. Barrier Membrane Material

**Figure 2:**
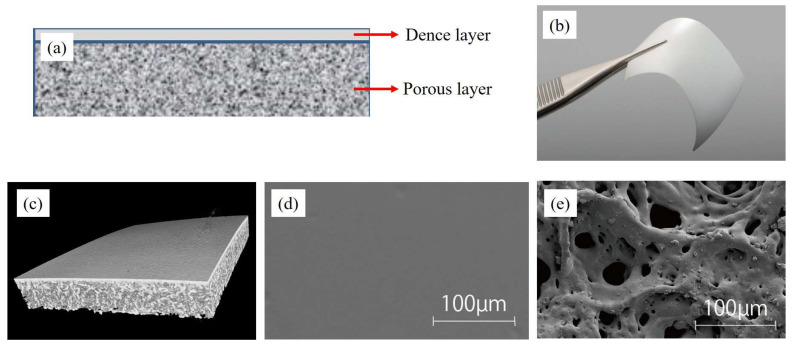
Barrier membrane material. A bilayer PLA/PCL membrane (Cytrans Elashield^®^, GC Corporation, Tokyo, Japan) has a white membrane structure molded from a bioabsorbable polymer. (**a**) Structural diagram. PLA/PCL consists of two layers, a dense layer and a porous layer. (**b**) PLA/PCL is a rectangular film measuring approximately 30 mm × 40 mm and has a thickness of approximately 200 μm. (**c**) CT image. (**d**,**e**) SEM image of a dense layer and a porous layer.

A bilayer PLA/PCL membrane (Cytrans Elashield^®^, GC Corporation, Tokyo, Japan) was used as the barrier membrane for the open window in lateral approach MSA. The PLA/PCL membrane has a white membrane structure molded from a bioabsorbable polymer. It is a rectangular film measuring approximately 30 mm × 40 mm and consists of two layers, a dense layer and a porous layer, with a thickness of approximately 200 μm. The dense layer is placed on the soft tissue side, and the porous layer is on the hard tissue side. The dense layer blocks fibroblast invasion, while the porous layer promotes osteoblast differentiation. The GC Corporation provided PLA/PCL (Figure 2) [24].

### 2.4. Surgical Protocol (SMA)

**Figure 3:**
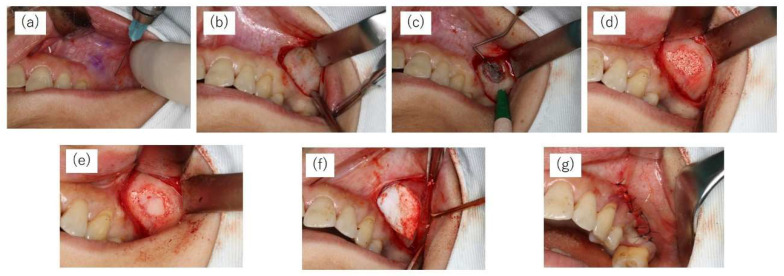
Surgical protocol of the maxillary sinus augmentation operation with lateral window technique. (**a**) Pre-operation. (**b**) A mucoperiosteal flap is created. (**c**) Dimensions of the minimally invasive lateral window. (**d**) After elevation of the basilar membrane of the sinus, the elevated space is filled with CO3Ap granules. (**e**) The bony window is repositioned. (**f**) A PLA/PCL membrane is placed over it. (**g**) The mucoperiosteal flap is sutured.

All patients were instructed to take an oral dose (1 g) of amoxicillin hydrate (Amoxicillin Capsules; Nichi–Iko Pharmaceutical Co., Tokyo, Japan) 1 h before surgery. MSA was conducted under local infiltration anaesthesia (2% lidocaine including 1/80,000 adrenaline). The bony window of the lateral maxillary wall was designed according to the planned location(s) of the implant(s) and the anatomy of the maxillary sinus. A horizontal incision was made on the buccal side of the alveolar bone at the maxillary molar defect site, and the mucoperiosteal flap was turned over to expose the lateral wall of the maxillary sinus. A small diamond bar was used to cut 360° around the entire circumference of the bone to form a bony window, which was separated from the sinus membrane. The mucosa of the maxillary sinus floor was carefully dissected and elevated using a mucosa elevator. The sinus floor mucosa was carefully checked for perforation. After elevation of the basilar membrane of the sinus, the elevated space was filled with CO3Ap granules. Finally, the bony window was repositioned; a PLA/PCL membrane was placed over it; and the mucoperiosteal flap was sutured (Figure 3). All maxillary sinus augmentations with the lateral approach were performed employing a staged approach.

To prevent postoperative infection, patients received amoxicillin 250 mg four times a day for five days and 0.2% benzethonium chloride mouthwash four times a day for two weeks, as well as loxoprofen sodium 60 mg three times a day for three days as an analgesic. Sutures were removed two weeks after surgery.

### 2.5. Radiographic Examinations

Cone beam computed tomography (CBCT) examinations were performed preoperatively (pre–MSA), within one week postoperatively (post–MSA1W), and at 5–6 months postoperatively (post–MSA5M), in accordance with the protocol shown in Figure 1. All images were obtained using the KaVo 3D eXam (KaVo Dental Systems, Biberach, Germany). The scanning parameters were 120 kVp, 5 mA, 8.9 s acquisition time, 0.25 mm thick axial slice and isotropic voxel size, and an imaging area of 16 cm × 16 cm.

### 2.6. Evaluation Items

**Figure 4:**
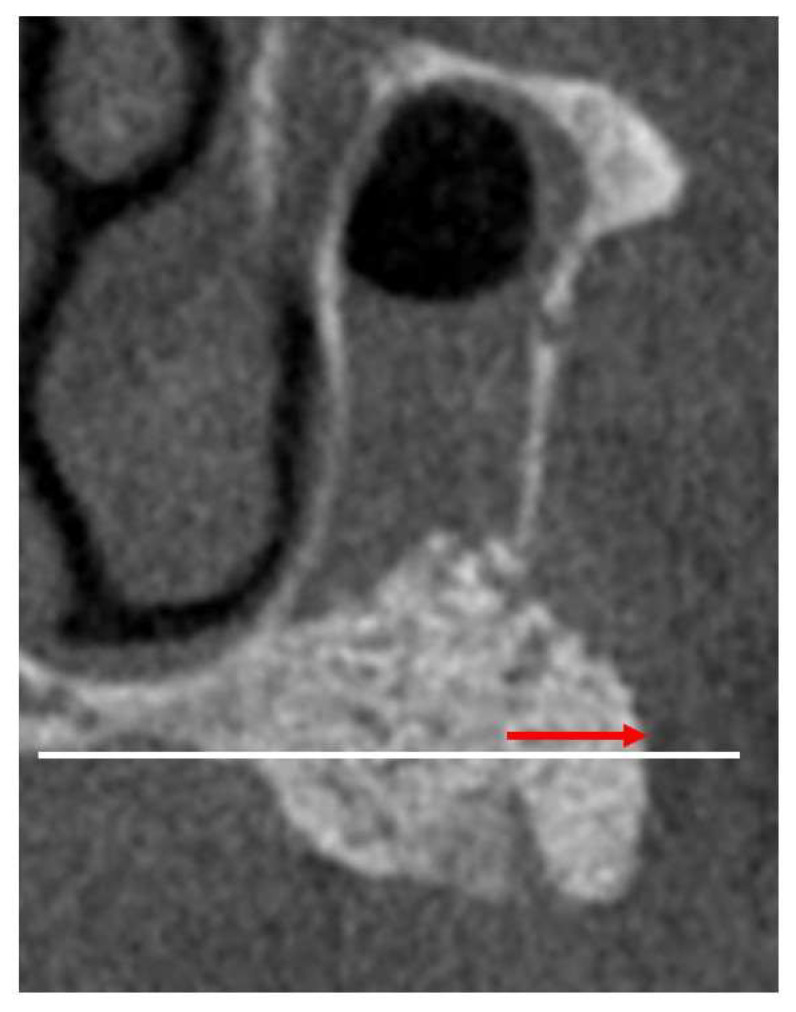
A CT scan showing the presence and amount of bone graft displacement (mm). An extended line connects the bilateral palatine bones (white line) in the coronal section at the lateral open window. The bone graft displacement was measured parallel to it (red arrow).

The following items were evaluated to measure the effectiveness of the PLA/PCL membrane in MSA: presence or absence of infection and sinusitis, exposure of barrier membrane, bone height post–MSA1W and post–MSA5M, rate of reduction of bone height post–MSA1W and post–MSA5M, presence and amount of bone graft displacement (mm; Figure 4).

Inflammation, inflection, and sinusitis were assessed by the presence or absence of persistent pain for at least one week postoperatively; the presence or absence of redness, swelling, and drainage of pus in the oral cavity; the condition of the maxillary sinus by CBCT two weeks postoperatively; and the patient’s nasal symptoms.

**Figure 5:**
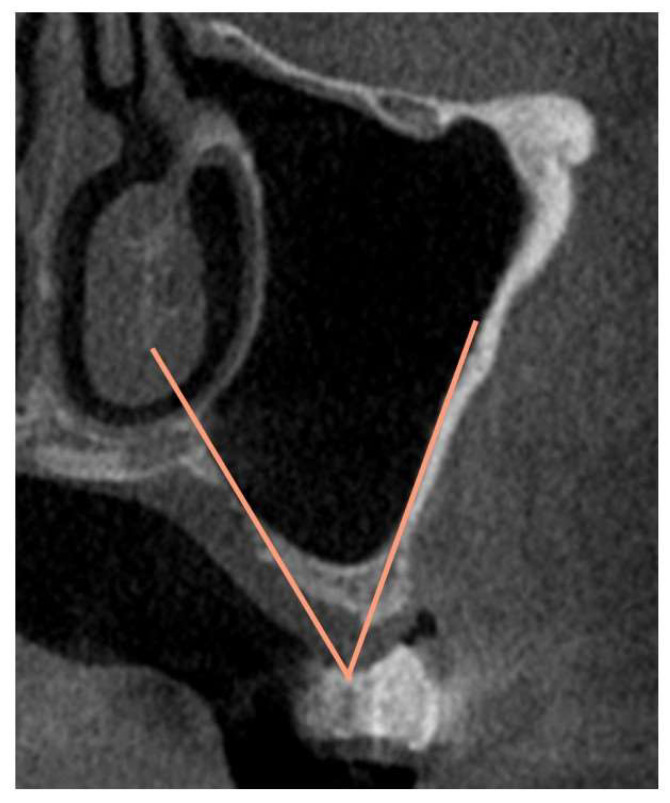
Maxillary sinus angle (between the lateral and medial wall of the maxillary sinus (orange line). Measurements were obtained within the plane perpendicular to the maxillary buccal bone of the tooth defect in the CT image.

**Figure 6:**
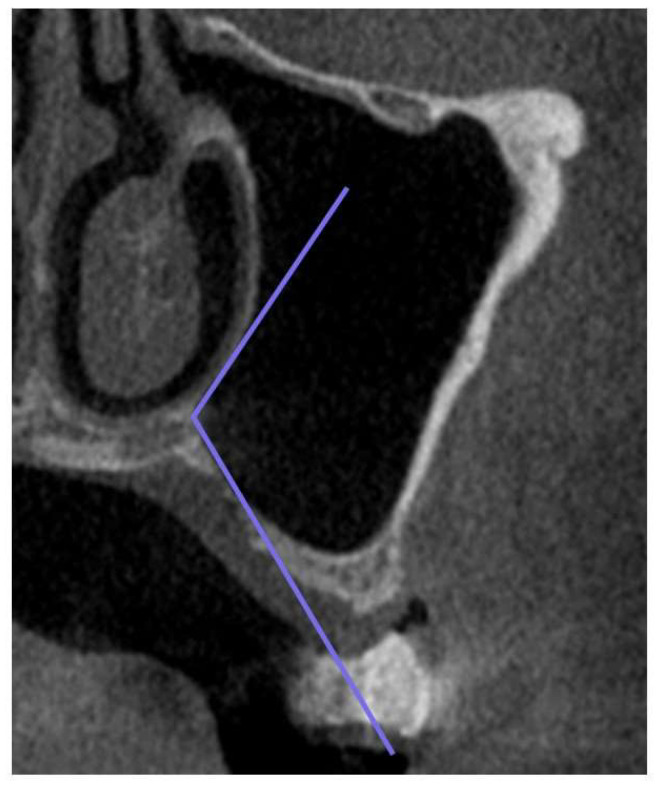
Palatonasal recess (PNR), between the roof of the hard palate and the lateral wall of the nasal cavity (purple line). Measurements were acquired within a plane perpendicular to the maxillary buccal bone of the tooth defect in the CT image.

**Figure 7:**
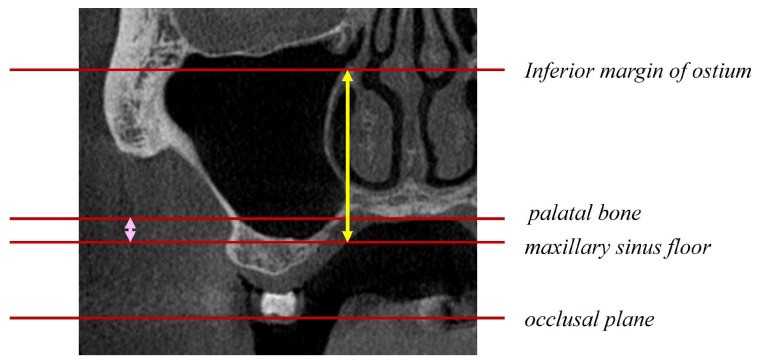
Alignment of the inferior margin of the ostium, palatine bone, and maxillary sinus floor as a straight line parallel to the occlusal plane. Distance from the maxillary sinus floor to the maxillary ostium (yellow arrow) and distance from the maxillary sinus floor to the palatal bone (pink arrow).

The following factors were evaluated concerning bone graft displacement and the percentage decrease in bone height (Figure 5, Figure 6 and Figure 7): sex; age; maxillary sinus angle (between the lateral and medial wall of the maxillary sinus: Figure 5); palatonasal recess (PNR, between the roof of the hard palate and the lateral wall of the nasal cavity: Figure 6); height from the maxillary ostium to the maxillary sinus floor (Figure 7, yellow marker); and height from the palatal bone to the maxillary sinus floor (Figure 7, pink marker).

The same oral and maxillofacial radiologist, with over 20 years of experience, performed all measurements.

### 2.7. Statistical Analysis

The effectiveness of the PLA/PCL membrane was evaluated using the Wilcoxon signed-rank test. The factors related to bone graft displacement and the percentage decrease in bone height were evaluated using the Mann–Whitney U test, Spearman’s correlation, and the chi-square test. Moreover, multiple comparisons among the three groups were performed using the Friedman test. In addition, *p*–values of <0.05 were considered statistically significant (IBM SPSS Statics; IBM, Tokyo, Japan).

## 3. Results

Table 2 shows the patient data. The study population included 24 patients (11 men, 13 women) aged 43–64 years (mean age, 53.5 ± 6.52 years). Bone graft displacement from the bony window was noted in eight patients (33.3%). Partial exposure of the barrier membrane occurred in two patients but without postoperative inflammation, infection, or maxillary sinusitis. Intraoperative sinus membrane perforation during MSA was not observed in any of the patients.

**Figure 8:**
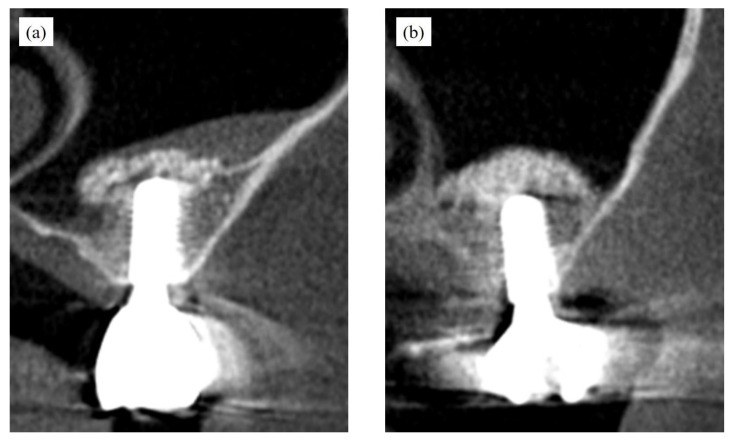
Implant placement and the prosthesis worn. (**a**) with bone displacement, and (**b**) without displacement.

Approximately 6–7 months after MSA, implants with a diameter of 4.1 mm and a length of 10 mm were placed in all cases, with or without bone displacement (Figure 8), and the final superstructures were inserted without problems.

**Figure 9:**
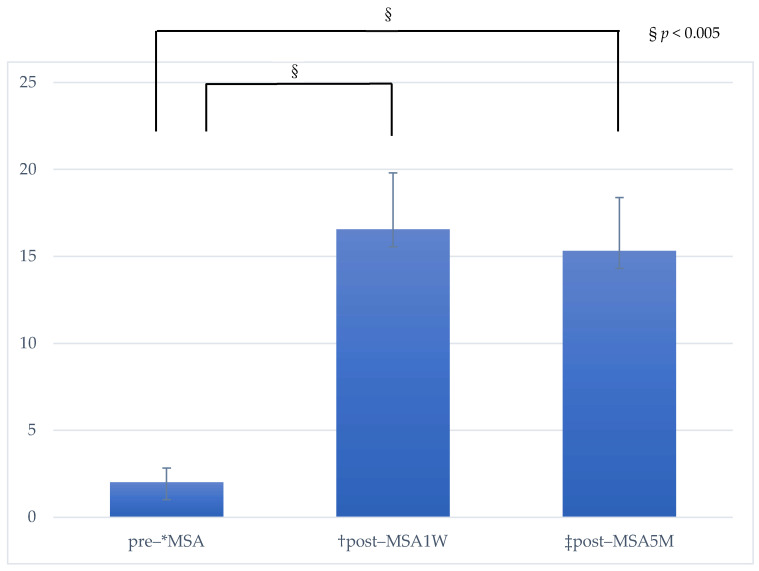
Changes in bone height. The measurement represents the height from the alveolar bone to the maxillary sinus floor. * MSA, maxillary sinus augmentation with a lateral approach. ^†^ Post–MSA1W, 1 week after MSA. ^‡^ Post–MSA5M, 5–6 months after MSA. ^§^ *p* < 0.005, Multiple comparisons among the three groups were performed using the Friedman test.

The mean preoperative existing bone height was 2.0 ± 0.8 mm, and the mean post–MSA bone height was 16.6 ± 3.3 mm post–MSA1W and 15.3 ± 3.1 mm post–MSA5M. Compared to the pre–MSA value, bone height increased significantly at post–MSA1W and post–MSA5M (*p* < 0.005). However, no difference was observed in bone height between post–MSA1W and post–MSA5M (Figure 9). Moreover, the reduction rate in maxillary sinus augmentation from post–MSA1W to post–MSA5M was 8.38% ± 4.88%.

### 3.1. Factors Related to Bone Graft Displacement

Factors related to bone displacement from the open window included sex, presence of septa, height from the maxillary sinus floor to the palatal bone, and height from the maxillary sinus floor to the maxillary ostium (Table 3).

Bone displacement was more likely in women than in men (*p* = 0.033), in those without a septum (*p* = 0.040), and in those with smaller distances from the maxillary sinus floor to the palatal bone and from the maxillary sinus floor to the maxillary ostium (*p* = 0.033 and *p* = 0.025, respectively).

### 3.2. Factors Associated with Reduced Augmentation (Table 4)

**Figure 10:**
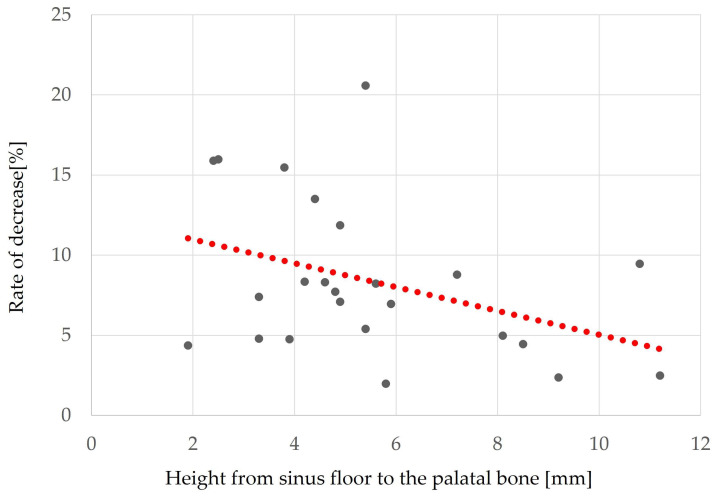
Relationship between height from sinus floor to the palatal bone and the rate of bone height reduction. The red dot line shows these correlations. A greater distance from the sinus floor to the palatal bone corresponds to a lower rate of decreased bone height (*p* = 0.032, r = −0.38).

**Figure 11:**
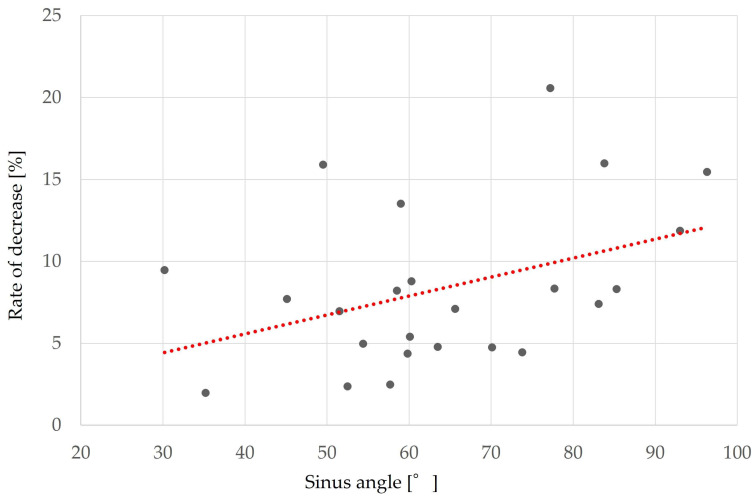
Relationship between the sinus angle and the rate of bone height reduction. The red dot line shows these correlations. A larger sinus angle corresponds to a larger rate of decreased bone height (*p* = 0.025, r = 0.41).

Factors related to reduced maxillary sinus augmentation were the height from the maxillary sinus floor to the palatal bone and the sinus angle. The smaller the height from the maxillary sinus floor to the palatal bone and the greater the sinus angle, the greater the decrease in augmentation (*p* = 0.032, r = −0.38 (Figure 10) and *p* = 0.025, r = 0.41 (Figure 11), respectively).

**Table 4:** Factors related to decreased maxillary sinus augmentation.

	Mean	*p* Value	r
Age	53.5 ± 6.5		
Sinus angle (°)	64.3 ± 17.0	0.025	0.41
Height from maxillary ostium to sinus floor (mm)	35.1 ± 4.1	0.49	
Height from sinus floor to palatal bone (mm)	5.5 ± 2.5	0.032	−0.38
* PNR (°)	117.2 ± 26.0	0.503	

* PNR, palatonasal recess between the roof of the hard palate and the lateral wall of the nasal cavity.

## 4. Discussion

The membrane used in the present study is a bioabsorbable polymer PLA/PCL with a simple composition and is already being used as a raw material in artificial dura mater grafts [25]. Clinical studies of maxillary sinus augmentation have reported that when the existing bone height is <4 mm in maxillary molar defects, maxillary sinus augmentation using the lateral window technique yields good clinical outcomes [3,21]. Notably, in two–stage maxillary sinus augmentation, placing a barrier membrane in the open window is essential to prevent connective tissue from penetrating into the grafted bone material and to maintain the elevation of the grafted bone. This clinical study aimed to evaluate the clinical usefulness of a PLA/PCL membrane as a barrier membrane in MSA by measuring the bone height and bone graft displacement from the open window after MSA.

Previous studies on the effectiveness of barrier membranes have evaluated MSA with various barrier membranes. In a meta–analysis, Suarez–Lopez del Amo et al. [26] reported that placing a barrier membrane in the open window did not affect the amount of vital bone formation for at least 6 months after surgery. Other studies [13,27,28] have reported that membrane use does not substantially increase vital bone volume. In contrast, Tarnow et al. [15] reported a significant increase in vital bone volume in the group using a barrier membrane (25.5%) compared to the group without a barrier membrane (11.9%), indicating a lack of consensus regarding the usefulness of barrier membranes. However, these studies did not adequately control for many relevant factors, including open window size [29], preoperative existing bone height [30], bone graft material, buccopalatal dimensions of the sinus cavity [31], and the biopsy collection site. For the biopsy collection site, the location and depth of the tissue sample may influence the mineralization and the degree of bone formation. Most clinical studies collected biopsy samples from the alveolar crest, whereas the barrier membranes are placed in the open window [32]. Therefore, previous studies may not have evaluated the true effects of barrier membranes.

In a systematic review and meta–analysis by Starch–Jensen et al. [32] on the presence or absence of a barrier membrane for MSA in the open window in the lateral wall, there was no statistically significant difference in implant survival rates after MSA with or without a barrier membrane. Although there was no statistically significant difference in implant survival rates after MSA with and without a barrier membrane, barrier membrane coverage increased newly formed bone by 6.4% and decreased proliferation of non–mineralized tissue by 1.1%. The conclusion is that barrier membrane coverage is beneficial and prevents displacement of the grafting material. Furthermore, Sim et al. [33], in an animal study using a sinus model with a collagen membrane placed against an open window, also found that the percentage of newly formed bone was greater in the groups with the collagen membrane than in the groups without. Augmentation volume was also significantly higher in the groups with collagen membrane than those without collagen membrane. Placement of a barrier membrane on the open window is effective.

In the present study, the mean bone height pre–MSA was 2.02 ± 0.81 mm, whereas the mean height post–MSA1W was 16.26 ± 3.09 mm and post-MSA5M was 15.05 ± 2.99 mm, indicating a mean reduction of 8.38% ± 4.88% in augmentation. In a similar study that did not use barrier membranes, Nakagawa et al. [34] investigated bone height immediately after MSA and at 7 ± 2 months by CBCT when using CO3Ap, a bone graft material similar to that used in our study. The bone height immediately after MSA was 13.3 ± 1.7 mm, and that at 7 ± 2 months after SFA was 10.7 ± 1.9 mm, a reduction of 19.27%. In a study comparing CO3AP to deproteinized bovine bone mineral (DBBM; Bio-Oss^®^, Geistlich Pharma AG, Wolhusen, Switzerland) as a control group that was similar to the present study but did not use barrier membranes, Nagata et al. [35] evaluated the bone graft volume reduction rate immediately after MSA and 6 months postoperatively, reporting rates of 14.2% for CO3AP and 25.2% for DBBM. Compared to studies that did not use barrier membranes, this study showed a significantly lower reduction in bone height (Table 5). Moreover, this study observed bone graft displacement in eight patients (33.3%). Few studies have examined bone graft displacement. Ohayon et al. [22] investigated the amount of bone displacement from open windows with and without collagen barrier membranes. They reported that bone graft displacement occurred in 76.5% of patients using a collagen barrier membrane. The PLA/PCL membrane was more flexible and less prone to tearing, which may have allowed it to fit into the open window and prevent bone graft displacement during the healing period, which was significantly less than with the collagen barrier membrane used in the study by Ohayon et al. The L-lactide–ε-caprolactone copolymer is a biologically safe material that does not inhibit bone formation and is a long-term resorbable membrane with a resorption period of 6 months, which was considered sufficient to prevent connective tissue invasion and promote bone regeneration. Therefore, the PLA/PCL membrane was effective as a barrier membrane in the open window of the MSA [24]; (see Table 6).

The results of our study also showed that the sinus angle and the height from the palatal bone to the maxillary sinus floor were factors related to reduced maxillary sinus elevation, with smaller distances from the palatal bone to the maxillary sinus floor and larger sinus angles being associated with larger decrease rates. Factors related to bone graft displacement included sex, presence of a septum, height from the maxillary sinus floor to the palatal bone, and height from the maxillary sinus floor to the maxillary ostium. Bone displacement was more likely to occur in women, those without a septum, and those with smaller distances from the palatal bone to the maxillary sinus floor and smaller distances from the maxillary sinus floor to the maxillary ostium. In the study by Ohayon et al. [22] on bone displacement, the authors stated that swelling of the maxillary sinus floor mucosa after MSA increases pressure in the sinus cavity and places physiological pressure on the bone graft material; consequently, if the open window is not covered with a membrane, the bone graft material will move toward the open window and even be displaced through it. They reported that postoperative bone graft displacement is associated with sinus membrane swelling, exacerbated by factors that increase sinus pressure, such as sniffing, sneezing, and bleeding. That pressure from sinus membrane swelling and bleeding can cause bone graft material to displace outside the sinus cavity. Furthermore, women were more at risk for bone graft displacement, which may be due to the effect of menopause on bone regeneration [36], as it has been reported that the volume of the maxillary sinus is significantly smaller in women than in men [37,38,39]. Many women in this study were in their 50s or older. Therefore, the results of the present study suggest that bone displacement from the open window is more likely to occur in women with a smaller volume and a smoother maxillary sinus without sinus septa and that this leads to a decrease in the amount of augmentation.

Limitations of this study include the lack of pathological evaluation and investigation, the lack of control for the position and size of the open window, and the lack of comparison with other membranes or other graft materials because this was a single-arm study. In particular, the position and size of the open window need to be thoroughly examined, including the results of this study, as there are reports of differences in augmented bone volume and the percentages of newly formed bone [33,40,41]. Furthermore, it is necessary to control for the size and position of the open window and the location of the pathology collection site, which have not been controlled in previous reports, when pathological evaluation is carried out. Long-term follow-up studies on the histopathological examination, the relationship with the anatomical morphology of the maxillary sinus, including the osteomeatal complex, and changes in bone volume after MSA and implant treatment are necessary.

## 5. Conclusions

Compared to other previously reported membranes, the PLA/PCL membrane could serve as a helpful barrier membrane for MSA. Factors related to bone graft displacement included sex, presence of a septum, height from the maxillary sinus floor to the palatal bone, and height from the maxillary sinus floor to the maxillary ostium. Therefore, focusing on these factors and the usual measurement points as a preoperative diagnosis with CT imaging would result in a higher quality MSA.

## Figures and Tables

**Table 1:** Inclusion and exclusion criteria.

**Inclusion criteria**
Residual alveolar bone height of less than 4 mm from the original sinus floor to the crest of the alveolar bone in * CT images
Age: between 20 and 65 years
Non-smoker
**Exclusion criteria**
Wearing a removable prosthetic
Respiratory diseases such as bronchial asthma and bronchitis
History of bone metabolism disease (e.g., osteoporosis) and related medication (e.g., bisphosphonate)
Alcohol and drug addiction, mental illness
Cysts, tumors, or inflammation in the maxillary sinus on * CT scan results
Undergoing or previously undergoing radiation therapy to the head and neck
Heart disease, blood disorders, collagen disease
Severe impairment of the kidney or dialysis
Pregnancy, possible pregnancy, breastfeeding, considering pregnancy
History of hypersensitivity or allergy to materials containing lactic/ε-caprolactone copolymers
Lifestyle, oral hygiene, or other factors that would make the patients ineligible for participation in the study

* CT, computed tomography.

**Table 2:** Patient data.

Sex	Male	11 (average age: 51.6 ± 8.0)
	Female	13 (average age: 55.1 ± 4.7)
Membrane exposure		2/24
Postoperative sinusitis		0/24
Bone graft displacement		8/24
Septa	Present	10/24
	Absent	14/24
Bone height (mm)	Pre– * MSA	2.0 ± 0.8
	^†^ Post–MSA1W	16.6 ± 3.3
	^‡^ Post–MSA5M	15.3 ± 3.1
Sinus angle (°)		64.3 ± 17.0
Height from maxillary ostium to sinus floor (mm)		35.1 ± 4.1
Height from sinus floor to palatal bone (mm)		5.5 ± 2.5
^§^ PNR (°)		117.2 ± 26.0
Preoperative sinus mucosal thickness (mm)		0.58 ± 1.1

* MSA, maxillary sinus augmentation with a lateral approach. ^†^ Post–MSA1W, 1 week after MSA. ^‡^ Post–MSA5M, 5–6 months after MSA. ^§^ PNR, palatonasal recess between the roof of the hard palate and the lateral wall of the nasal cavity.

**Table 3:** Factors related to bone graft displacement.

			Displacement		*p* Value
			Present	Absent	
Sex		Male	1	10	0.033
		Female	7	6	
Septa		Presence	1	9	0.040
		Absence	7	7	
			**Mean**		
Bone height (mm)	Pre– * MSA		2.1 ± 0.9	2.0 ± 0.8	0.87
	^†^ Post–MSA1W		16.3 ± 3.0	16.7 ± 3.5	
	^‡^ Post–MSA5M		15.1 ± 3.0	15.4 ± 3.2	
Sinus angle (°)			69.0 ± 16.4	61.9 ± 17.3	0.147
Height from maxillary ostium to sinus floor (mm)			32.8 ± 3.4	36.3 ± 4.1	0.025
Height from sinus floor to palatal bone (mm)			4.0 ± 1.0	6.3 ± 2.7	0.033
^§^ PNR(°)			110.5 ± 31.1	120.5 ± 22.4	0.47

* MSA, maxillary sinus augmentation with a lateral approach. ^†^ Post–MSA1W, 1 week after MSA. ^‡^ Post–MSA5M, 5–6 months after MSA. ^§^ PNR, palatonasal recess between the roof of the hard palate and the lateral wall of the nasal cavity.

**Table 5:** Reduction in bone height from a week to 5–6 months postoperatively.

Nagata et al. [35]		Nakagawa et al. [34]	This Study	*p* Value
* CO3AP 14.2%	^†^ DBBM 25.2%	19.27%	8.38% ± 4.88%	2.4 × 10^−4 ‡^

* CO3AP, carbonate apatite. ^†^ DBBM, deproteinized bovine bone mineral. ^‡^ *p* < 0.001, compared to the data of Nakagawa et al. by Mann–Whitney U test; statistically significant difference.

**Table 6:** Rate of bone graft displacement.

Ohayon et al. [22]	This Study	*p* Value
Presence: 13/17 patients (76.5%)	Presence: 8/24 patients (33.3%)	2.9 × 10^−5^ *

* *p* < 0.001, compared to the result of Ohayon et al. by Mann–Whitney U test; statistically significant difference.

## Data Availability

The datasets used and analyzed during the current study are available from the corresponding author on reasonable request.
